# Identification of a novel mechanism of action of fingolimod (FTY720) on human effector T cell function through TCF-1 upregulation

**DOI:** 10.1186/s12974-015-0460-z

**Published:** 2015-12-30

**Authors:** Maria Antonietta Mazzola, Radhika Raheja, Gopal Murugaiyan, Hasan Rajabi, Deepak Kumar, Thomas Pertel, Keren Regev, Russell Griffin, Lilian Aly, Pia Kivisakk, Parham Nejad, Bonny Patel, Nguendab Gwanyalla, Hillary Hei, Bonnie Glanz, Tanuja Chitnis, Howard L. Weiner, Roopali Gandhi

**Affiliations:** Brigham and Women’s Hospital, Ann Romney Center for Neurologic Diseases, Harvard Medical School, 77 Avenue Louis Pasteur, Boston, MA 02115 USA; Dana Farber Cancer Institute, Boston, MA 02115 USA; Department of Biochemistry and Molecular Pharmacology, Harvard Medical School, Boston, MA 02115 USA; Partners MS Center, Brigham and Women’s Hospital, 1 Brookline Place, Brookline, MA 02445 USA

**Keywords:** Multiple sclerosis, Fingolimod (FTY720), TCF-1, IFN-γ, Granzyme B

## Abstract

**Background:**

Fingolimod (FTY720), the first oral treatment for multiple sclerosis (MS), blocks immune cell trafficking and prevents disease relapses by downregulation of sphingosine-1-phosphate receptor. We determined the effect of FTY720 on human T cell activation and effector function.

**Methods:**

T cells from MS patients and healthy controls were isolated to measure gene expression profiles in the presence or absence of FTY720 using nanostring and quantitative real-time polymerase chain reaction (qPCR). Cytokine protein expression was measured using luminex assay and flow cytometry analysis. Lentivirus vector carrying short hairpin RNA (shRNA) was used to knock down the expression of specific genes in CD4+ T cells. Chromatin immunoprecipitation was performed to assess T cell factor 1 (TCF-1) binding to promoter regions. Luciferase assays were performed to test the direct regulation of interferon gamma (IFN-γ) and granzyme B (GZMB) by TCF-1. Western blot analysis was used to assess the phosphorylation status of Akt and GSK3β.

**Results:**

We showed that FTY720 treatment not only affects T cell trafficking but also T cell activation. Patients treated with FTY720 showed a significant reduction in circulating CD4 T cells. Activation of T cells in presence of FTY720 showed a less inflammatory phenotype with reduced production of IFN-γ and GZMB. This decreased effector phenotype of FTY720-treated T cells was dependent on the upregulation of TCF-1. FTY720-induced TCF-1 downregulated the pathogenic cytokines IFN-γ and GZMB by binding to their promoter/enhancer regions and mediating epigenetic modifications. Furthermore, we observed that TCF-1 expression was lower in T cells from multiple sclerosis patients than in those from healthy individuals, and FTY720 treatment increased TCF-1 expression in multiple sclerosis patients.

**Conclusions:**

These results reveal a previously unknown mechanism of the effect of FTY720 on human CD4+ T cell modulation in multiple sclerosis and demonstrate the role of TCF-1 in human T cell activation and effector function.

**Electronic supplementary material:**

The online version of this article (doi:10.1186/s12974-015-0460-z) contains supplementary material, which is available to authorized users.

## Background

Multiple sclerosis is a chronic inflammatory disorder of the central nervous system (CNS) driven by autoreactive lymphocytes that induce an inflammatory cascade leading to damage of myelin and axon, resulting in neurodegeneration [1]. In particular, T cells that produce pro-inflammatory cytokines such as interferon gamma (IFN-γ) and interleukin (IL)-17 play a critical role in multiple sclerosis pathogenesis [1–8], and reduced levels of these effector molecules are associated with better therapeutic responses [9–11]. Although the expression of granzyme B (GZMB) is linked to the pathogenic signature of T cells in experimental autoimmune encephalomyelitis (EAE) [12] and multiple sclerosis [13], its precise role in multiple sclerosis is still under investigation.

Fingolimod (FTY720), a sphingosine 1-phosphate (S1P) receptor modulator, was approved as the first oral treatment for multiple sclerosis based on results of three separate clinical trials among patients with relapsing-remitting multiple sclerosis (RRMS) [14–16]. S1P receptors are highly expressed on membranes of lymphocytes and are critical for T and B cell egress from secondary lymphoid organs. The phosphorylated form of FTY720 causes internalization and degradation of S1P receptors, resulting in the retention of lymphocytes in lymph nodes [17]. FTY720 primarily reduces the number of naïve T cells and central memory T cells in the circulation due to their expression of homing receptor CCR7 [18].

T cell factor 1 (TCF-1), also known as *TCF7* (gene name), is a transcription factor present in hematopoietic T cells that has an important function in T cell development in the thymus. TCF-1 negatively regulates Th1 [19] and Th17 [20, 21] differentiation while promoting Th2 differentiation, via stimulation of GATA3 (a Th2-specific transcription factor) [19]. *TCF7* knock-out mice are susceptible to EAE [20] and develop aggressive T cell deficiencies resembling human T cell acute lymphoblastic leukemia [22]. Interestingly, a computational re-analysis of multiple sclerosis-associated single nucleotide polymorphism data from 112 different cell types suggests that *TCF7* is associated with multiple sclerosis [23], and a recent genome-wide association study identified the single nucleotide polymorphism rs756699 located on the *TCF7* gene in multiple sclerosis patients [24]. However, the role of TCF-1 in the regulation of human CD4+ T cell effector function and its relevance to multiple sclerosis and treatment response are unknown.

In this study, we found that FTY720 modulates CD4+ T cell activation and effector function through TCF-1. FTY720-induced TCF-1 regulates the expression of IFN-γ and GZMB in T cells. Furthermore, T cells from multiple sclerosis patients exhibit lower *TCF7* expression than those from healthy individuals, and FTY720 treatment upregulates *TCF7* expression in T cells from both healthy controls and patients. Our findings establish that TCF-1 expression in human CD4+ T cells is linked to multiple sclerosis and that treatment with FTY720 increases TCF-1 expression, which regulates IFN-γ and GZMB production.

## Methods

### Subjects and blood samples

Peripheral venous blood was collected after obtaining informed consent from healthy individuals and multiple sclerosis patients. All patients were seen at the Partners Multiple Sclerosis Center at Brigham and Women’s Hospital. We included untreated RR multiple sclerosis patients and patients treated with FTY720 before and after 3 months of treatment. Patients were classified based upon their clinical characteristics as defined by 2010 Revisions to the McDonald Criteria [25] with the help of trained neurologists. Untreated multiple sclerosis patients had received no treatment with glatiramer acetate or interferons in the past 3 months, no treatment with other disease-modifying therapy in the past 6 months, and no steroids in the past month. Detailed characteristics of these patients are shown in Additional file 1: Table S1. Blood samples were collected under the Comprehensive Longitudinal Investigation of Multiple Sclerosis at Brigham and Women’s Hospital (CLIMB). This study was conducted in accordance with the WMA Declaration of Helsinki regarding ethical principles for medical research involving human subjects. The Partners Human Research Committee/Instutional Review Board approved the use of human material (IRB protocols 1999P010435/BWH and 2012P000394).

### Naïve CD4+ T cell isolation, culture, and flow cytometry analysis

Peripheral blood mononuclear cells (PBMCs) were isolated by Ficoll-Hypaque density gradient centrifugation (Pharmacia LKB Biotechnology, Piscataway, NJ). Naïve T cells from PBMCs were isolated using a Miltenyi Biotec (Alburn, CA) negative selection kit. Purified naïve CD4+ T cells were activated with plate-bound anti-CD3 (5 μg/ml, BD Bioscience, San Jose CA), soluble anti-human CD28 (1 μg/ml, BD Bioscience), and IL-2 (20 ng/ml, R&D Systems) with or without FTY720 (100 ng/ml, Novartis). After 6 days, cell-free culture supernatants were collected for cytokine analysis by Luminex assay (Miltenyi Biotec), and cells were harvested for RNA extraction and intracellular staining. Naïve T cells were stimulated with PMA (Sigma), ionomycin (Sigma), and Golgistop for 4 h. Cells were stained for anti-human CD4 APC (BD Bioscience) and violet fluorescent reactive dye VVD (Life Technologies), and then cells were fixed and permeabilized with BD fixation and permeabilization buffer and stained for IFN-γ FITC and GZMB FITC (BD Bioscience). For surface staining, the following antibodies were used: anti-human CD4 pacific blue, anti-human CCR7 PE, and anti-human CD45RA APC, IgG2a PE isotype control, IgG2b, and k APC isotype control (all from BD Bioscience). All antibodies were titrated for flow cytometry, which was performed on a BD LSR II (BD Bioscience) and analyzed using Flowjo software.

### Nanostring and quantification by real-time PCR

Total RNA was isolated from cultured T cells using RNA isolation kit (Norgen Biotek). RNA expression of 500 immune genes was detected by NanoString array (nCounter, Gene expression code set, Human Immunology Kit). Experiment was performed and analyzed following the manufacturers’ instructions. Data were normalized to spiked positive controls and housekeeping genes (nSolver Analysis system). Results from NanoString arrays were validated using Taqman PCR (Life Technologies). Briefly, first-strand cDNA synthesis was performed for each RNA sample from 0.5 to 1 μg of total RNA using Taqman reverse transcription reagents (Applied Biosystems). Quantitative real-time polymerase chain reaction (qPCR) was performed using primers for TCF-7 (Hs00175273_m1), IFNG (Hs99999041_m1), and GZMB (Hs001554355_m1) and the housekeeping genes GAPDH (Hs02758991_g1), G6PD (Hs00166169_m1), GUSB (Hs00939627_m1), TPB (Hs00427620_m1), TUBB (Hs00742828_s1), POLR1B (Hs00219263_m1), POLR2A (Hs00172187_m1), HPRT1 (Hs02800695_m1), PRL19 (Hs02338565_gH), EEFG1 (Hs01922638_u1), ALAS1 (Hs00963534_m1), and OAZ1 (Hs00427927_m1) with TaqMan Fast Universal qPCR Master Mix (No ampErase Uracil *N*-Glycosylase, Life Technologies). Samples were run on QuantStudio 7 (Applied Biosystems, Life Technologies). The mean of the 12 housekeeping genes was used as an endogenous control to normalize total cDNA for each sample, and all values are shown as relative expression.

### In vitro knockdown with shRNA

The expression of TCF-1 was knocked down in primary naïve CD4+ T cells by lentivirus vector carrying short hairpin RNA (shRNA) against TCF-1 (mission shRNA bacterial glycerol stock TRCN0000281336, Sigma-Aldrich) or a non-targeting sequence (a gift from Dr. Thomas Pertel at BWH and Dr. David Root at Broad Institute) as a control. Primary naïve CD4+ T cells were cultured in a 24-well plate in the presence of anti-CD3, anti-CD28, and IL2 and incubated with lentivirus. After 3 days, the lentivirus was washed away, and puromycin was added (1.5 μg/ml) to select transduced T cells for 3 days. Live cells were sorted using intracellular fluorescence-activated cell sorting, and the expression of TCF-1 was measured using qPCR.

### Chromatine immune precipitation assay

The binding of TCF-1, H3-Lys9, and H3-Lys27 on *IFNG* and *GZMB* were analyzed using chromatin immunoprecipitation (ChIP) assay as described previously [26, 27]. The antibodies used for ChIP assay were anti-TCF-1 (Cell Signaling), anti-trimethyl-histone H3-Lys9 (Millipore), and anti-trimethyl-histone H3-Lys27 antibodies (Millipore) or Rabbit IgG (Millipore). Input DNA and DNA recovered after immunoprecipitation were analyzed by real-time qPCR using primer pairs for *IFNG* and *GZMB* (*IFNG*: forward 5′-GAAGAGTCAACATTTTACCAGGGC and reverse 5′-GTGACAGATAGGCAGGGATGATAG; *GMZB*: forward 5′-GAACCTGGTGCAATTACCAGAAT and reverse 5′CTTTTCACAGGGATAAACTGCTGG) with SyBR green Fast Master mix (Applied Biosystems). Values for TCF-1 binding with *IFNG* promoter and *GZMB* enhancer regions were normalized to IgG.

### Luciferase reporter assay

HEK293T cells were maintained in DMEM medium (Gibco) supplemented with 10 % fetal bovine serum (Gibco), 4 mM L-glutamine (Lonza), 1 mM sodium pyruvate (Lonza), 1 % non-essential amino acid (Lonza), and 10 mM HEPES (Lonza) at 37 °C/5 % CO_2_. Forty-eight hours prior to transfection, cells were seeded at 10,000 cells per well in a 96-well tissue culture plate (Perkin Elmer). Cells were transfected with the indicated amounts of each expression vector using Lipofectamine 2000 (Invitrogen) following the manufacturer’s instructions. Renilla luciferase vector (12.5 ng) was used for normalization of transfection efficiency. Forty-eight hours after transfection, cells were assayed using the Dual-Luciferase Reporter Assay System kit (Promega) as per the manufacturer’s instructions. Firefly luciferase values were normalized to Renilla luciferase levels. The results were expressed as relative luciferase activity in relative light units (RLU). Transfections were carried out in triplicate in three independent experiments, and results are expressed as mean ± standard error.

### Plasmids

The −3.6-kb human IFNG-luc in a pGL3 vector background was a gift from Howard Young (Addgene plasmid #17599) [28], pcDNA3-HA-TCF1 was a gift from Kai Ge (Addgene plasmid #40620) [29], plasmid encoding Tbet was a gift from Dr. Christine Campbell [30], 1436 pcDNA3 Flag HA was a gift from William Sellers (Addgene plasmid #10792), and pGL3 basic and pGL3 promoter vector were purchased from Promega. A 1.04-kb fragment of the human GZMB enhancer region was inserted as three repeats between KpnI and NheI sites into the pGL3 promoter vector to generate the GZMB-luc plasmid (GenScript).

### Western blot

Naïve T cells were isolated from PBMCs, activated in the presence or absence of FTY720, and collected after 15, 30, 60, or 120 min. Cells were lysed with RIPA assay buffer (Thermo Scientific) supplemented with protease and phosphatase inhibitors (Thermo Scientific). Total protein concentration was determined by BCA assay (Thermo Scientific). Samples were prepared with 20 μg of protein, loading buffer (Life Technologies), and reducing agent (Life Technologies) and then heated for 10 min at 70 °C before use. Samples were run on a 10 % bis-tris gel (NOVO, Life Technologies), transferred to PVDF membrane, and detected by immunoblot. The following antibodies were used: p-AKT (Cell Signaling Technology), p-GSK3β (Cell Signaling Technology), pan-AKT (Cell Signaling Technology), pan-GSK3β (Cell Signaling Technology), beta actin (Cell Signaling Technology), and anti-rabbit IgG HRP (Cell Signaling Technology). The membrane was blocked with 5 % bovine serum albumin followed by primary and secondary antibody incubations as per the manufacturers’ instructions. Immunoblots were developed using ECL Prime (GE Healthcare).

### Statistical analysis

All statistical analyses were performed using GraphPad Prism software. Paired and unpaired non-parametric *t* tests were used to test for group differences. Correlations were calculated using Spearman’s rank correlation coefficient.

## Results

### FTY720 treatment decreases circulating CD4+ T cells and increases *TCF7* expression in CD4+ T cells.

FTY720 decreases relapses in multiple sclerosis, and its therapeutic effects are attributed to the sequestration of lymphocytes into the lymph nodes [18]. We characterized the frequency of total and naïve CD4+ T cells in multiple sclerosis patients before and 3 months after FTY720 treatment. Consistent with previous observation, [18] FTY720 treatment reduced the percentage and absolute number of circulating total CD4+ (Additional file 2: Figure S1 A and C) and naïve CD4+ T cells (Additional file 2: Figure S1 B and D), 3 months after treatment relative to baseline measurements. FTY720-treated patients continue to generate an immune response against foreign antigens indicating that the T cells still have the ability to egress from lymph nodes and potentially impact the CNS [31]. To further define the impact of FTY720 on immune cell function, we assessed the effect of FTY720 treatment on CD4+ T cell effector function.

**Fig. 1:**
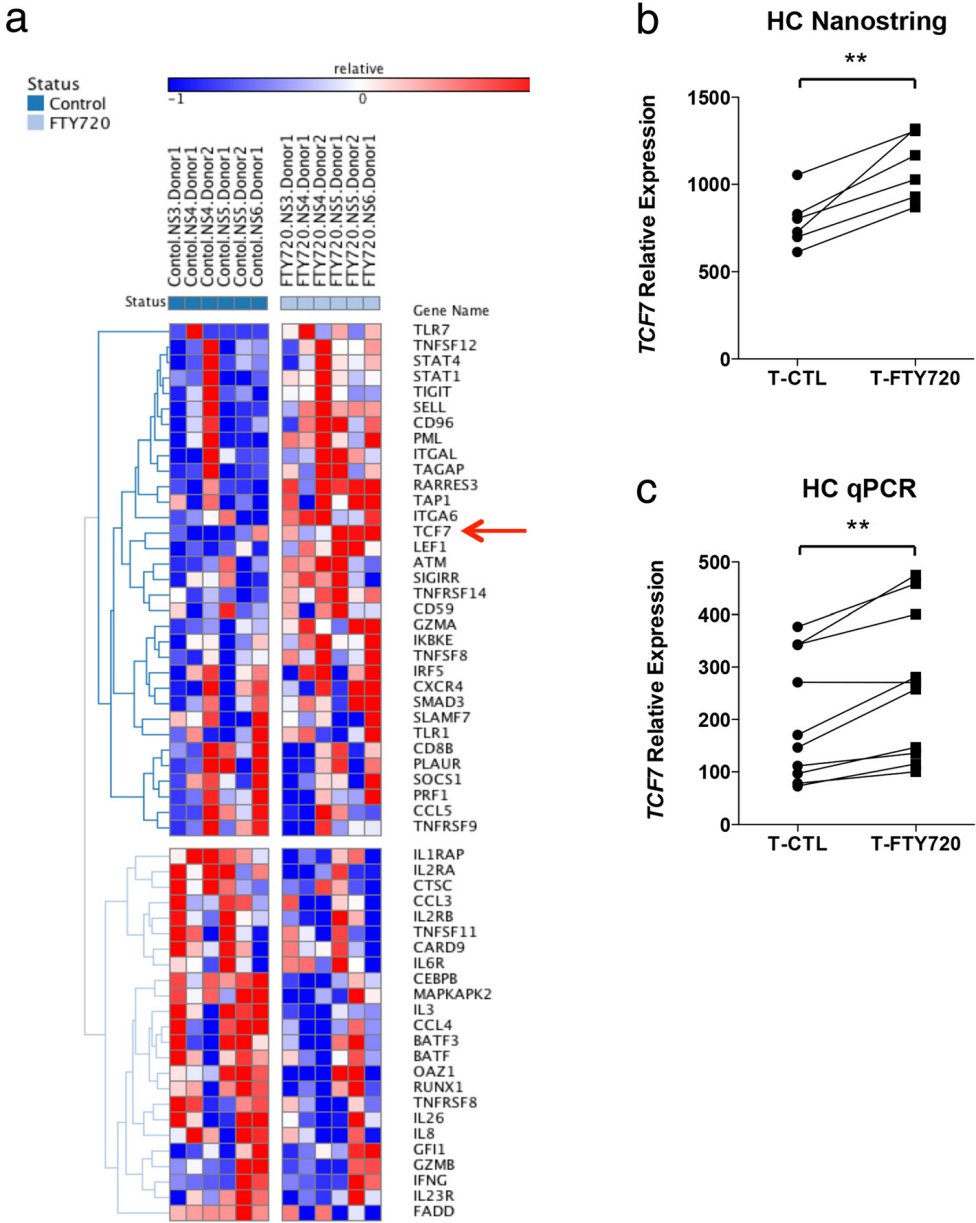
FTY720 treatment increases *TCF7* expression in T cells. **a** Heatmap showing significantly changing mRNA expression upon CD4 naïve T cell activation in the presence or absence of FTY720 as measured by a nanostring array of 500 immune genes. **b** Expression of *TCF7* in in vitro activated T cells from healthy control (HC) individuals in the presence (T-FTY720) or absence (T-CTL) of FTY720 analyzed using nanostring (*n* = 6). **c** Expression of *TCF7* in in vitro activated T cells from HC individuals in the presence or absence of FTY720 analyzed using qPCR (*n* = 10). **p < 0.01, paired non-parametric *t* tests

Purified naïve T cells from healthy individuals were activated in the presence or absence of FTY720 at concentration of 100 ng/ml. Although the physiological concentration of FTY720 in blood is in a range of 5 to 10 ng/ml [18, 32], the concentration of the lymph nodes is 10 times higher [33]. Since we wanted to recapitulate the physiological effect of FTY720 on T cell activation within the lymph nodes, we chose to use the concentration of 100 ng/ml. We measured the effect of FTY720 on T cell-associated immune gene expression using nanostring-based immune arrays. We found that FTY720-treated T (T-FTY720) cells had a distinct immune gene signature, with 33 upregulated genes and 24 downregulated genes relative to untreated T (T-CTL) cells (Fig. 1a). We selected *TCF7* based upon its relevance shown by several studies highlighting its role on T cell differentiation in mice and in EAE induction. In addition, recent observations in GWAS studies have correlated *TCF7* with MS pathogenesis. These studies, however, did not address the functional role of *TCF7* in MS patients, thus, we focused our attention on *TCF7*. We found that T-FTY720 cells showed greater *TCF7* expression than T-CTL cells (Fig. 1b). These results were further confirmed by qPCR, which also showed increase in the expression of *TCF7* in T-FTY720 compared to T-CTL (Fig. 1c). To determine whether this increase in *TCF7* expression was specifically brought about by FTY720, we activated T cells in the presence of other approved drugs for multiple sclerosis treatment, namely interferon beta and natalizumab. We found increased expression of *TCF7* only in T-FTY720 cells but not T cells treated with interferon beta (T-IFN beta) or natalizumab (T-Ty) (Additional file 3: Figure S2), suggesting that expression of *TCF7* is specifically enhanced upon FTY720 treatment.

### FTY720 treatment decreases effector T cell function

**Fig. 2:**
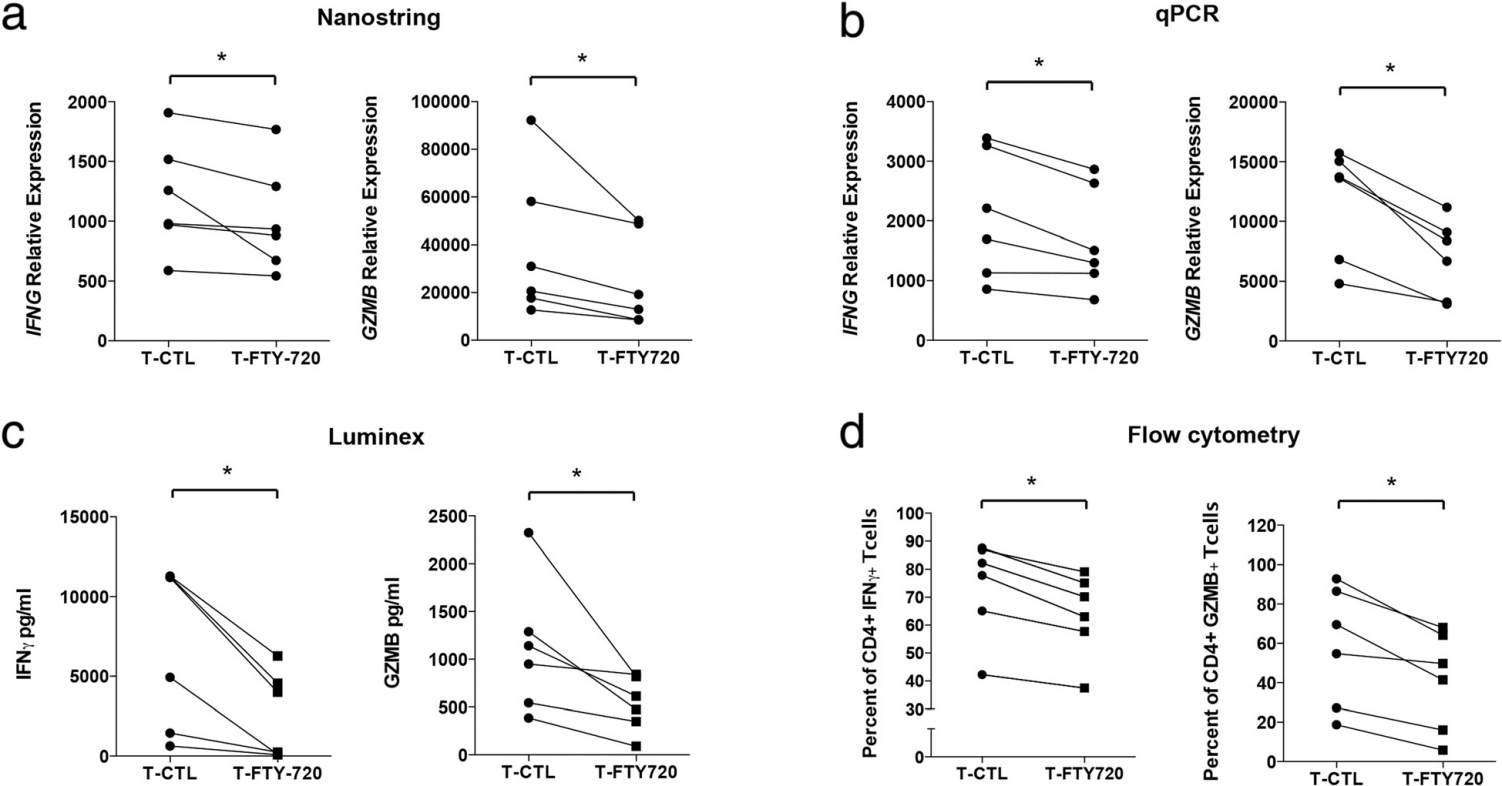
FTY720 treatment decreases effector T cell function. **a** Expression of *IFNG* and *GZMB* in in vitro activated T cells from healthy individuals in the presence or absence of FTY720 as determined by nanostring (*n* = 6). **b** Expression of *IFNG* and *GZMB* in in vitro activated T cells from healthy individuals in the presence or absence of FTY720 as determined by qPCR (*n* = 6). **c** Expression of IFN-γ and GZMB in in vitro activated T cells from healthy individuals in the presence or absence of FTY720 as determined by Luminex-based assay (*n* = 6). **d** Expression of IFN-γ and GZMB in in vitro activated T cells from healthy individuals in the presence or absence of FTY720 using flow cytometry (*n* = 6). *p < 0.05, **p < 0.01, paired non-parametric *t* tests

To investigate whether FTY720 modulates effector function of CD4+ T cells, we measured cytokine expression in naïve CD4+ T cells treated with or without FTY720. We observed that, compared to T-CTL cells, T-FTY720 cells showed lower messenger RNA (mRNA) expression of *IFNG* and *GZMB* as measured by nanostring array (Fig. 2a) and qPCR (Fig. 2b) and lower protein expression of IFN-γ and GZMB as measured by Luminex-based assay (Fig. 2c) and intracellular fluorescence-activated cell sorting analysis (Fig. 2d).

In addition to IFN-γ and GZMB expressions, we measured the expression of pro-inflammatory cytokines IL-17, granulocyte macrophage colony-stimulating factor (GMCSF), and tumor necrosis factor alpha (TNF-α) in supernatant collected from T-FTY720 and T-CTL cells using Luminex-based assay. We found decreased expression of IL-17, GMCSF, and TNF-α in T-FTY720 cells compared to T-CTL cells (Additional file 4: Figure S3 A). To determine whether this decreased cytokine production was related to excessive cell death, we measured the percentage of dead cells in T cell cultures in the presence or absence of FTY720. We found no difference in the percentage of dead T cells between groups (Additional file 4: Figure S3 B), suggesting that the effect of FTY720 is not mediated by increased cell death.

### TCF-1 regulates IFN-γ and GZMB expression

**Fig. 3:**
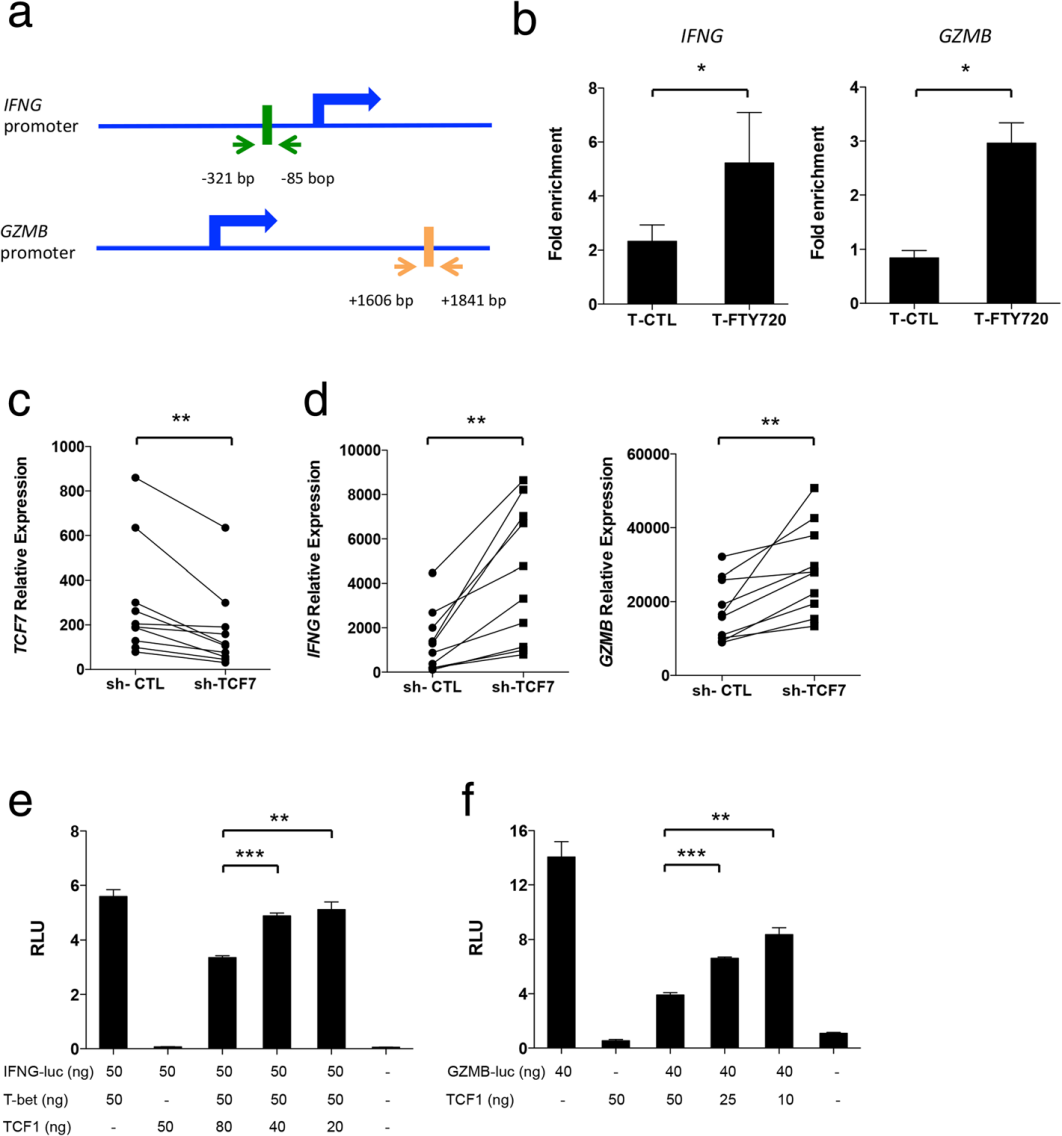
TCF-1 regulates *IFNG* and *GZMB* expression. **a** Binding region of TCF-1 in the promoter of *IFNG* and enhancer of *GZMB*. Blue arrows represent transcription start site. **b** qPCR analysis of chromatin-immunoprecipitated T cells showing interaction of TCF-1 with the *IFNG* promoter and *GZMB* enhancer region in primary T cells treated with or without FTY720 (*n* = 7). Data are shown as mean ± SEM. *p < 0.05, paired non-parametric *t* tests. **c** qPCR analysis of *TCF7* expression in T cells treated with *TCF7* shRNA (sh-TCF7) or negative control (sh-CTL). **p < 0.01, paired non-parametric *t* tests. **d** qPCR analysis of *IFNG* and *GZMB* expression in T cells treated with *TCF7* shRNA or negative control. **p < 0.01, paired non-parametric *t* tests. **e** Luciferase activity in HEK293T cells transfected with plasmids encoding firefly luciferase downstream of the *IFNG* promoter region (IFNG-luc), Tbet, TCF-1, or empty vector control. **f** Luciferase activity in HEK293T cells transfected with plasmids encoding firefly luciferase downstream of the *GZMB* enhancer region (GZMB-luc), TCF-1 or empty vector control. *RLU* relative light units. Data are shown as mean ± SEM. * **p < 0.01, ***p < 0.001, unpaired non-parametric *t* tests

TCF-1 regulates T cell development and differentiation in mice [19–21, 34–36]. However, the role of TCF-1 in the regulation of CD4+ T cell function in humans remains unclear. Therefore, we analyzed the transcriptional regulation of *IFNG* and *GZMB* in CD4+ T cells by TCF-1 upon FTY720 treatment. We performed an in silico analysis and found that TCF-1 has binding sites in the *IFNG* promoter (−321 to −85 bp) and *GZMB* enhancer (+1606 to +1841 bp) regions (Fig. 3a). Using chromatin immunoprecipitation (ChIP) assay, we found increased binding of TCF-1 on both *IFNG* promoter and *GZMB* enhancer regions in FTY720-treated cells (Fig. 3b).

Regulation of IFN-γ and GZMB expression by TCF-1 was investigated by knockdown of *TCF7* expression using lentivirus particles carrying shRNA against *TCF7* or negative control. We found that shRNA-mediated knockdown of *TCF7* (Fig. 3c) resulted in an increased *IFNG* and *GZMB* expression in T cells suggesting that TCF-1 might directly affect IFNG and GZMB expression (Fig. 3d).

Next, we performed a dual-luciferase reporter assay in HEK293T cells to determine whether TCF-1 directly represses IFN-γ and GZMB expression by binding to their promoter and enhancer regions, respectively. We used a plasmid encoding firefly luciferase downstream of the *IFNG* promoter region (IFNG-luc) that included the putative TCF-1 binding site determined by the ChIP assay as well as a binding site for Tbet, a transcription factor that reportedly activates IFN-γ expression [30, 37, 38]. We found increased relative firefly luciferase levels in cells co-transfected with IFNG-luc and Tbet expression plasmid compared with cells transfected with an empty vector. However, cells co-transfected with TCF-1 expression plasmid and IFNG-luc did not induce luciferase reporter activity; however, when both Tbet and TCF-1 expression plasmids were co-transfected with IFNG-luc, Tbet-induced firefly luciferase activity was reduced in a TCF-1 dose-dependent manner (Fig. 3e), suggesting that TCF-1 represses transcription at the *IFNG* promoter. We used a reporter plasmid containing the 1.04-kb *GZMB* enhancer sequence identified from our ChIP assay at a site upstream of the SV40 promoter that regulates firefly luciferase (GZMB-luc). We observed that firefly luciferase activity decreased significantly when HEK293T cells were co-transfected with GZMB-luc and increasing amounts of the TCF-1 expression plasmid (Fig. 3f). Firefly luciferase activity was unchanged in cells co-transfected with GZMB-luc reporter plasmid and an empty vector suggesting that repression of luciferase activity was TCF-1 dependent.

Using in silico analysis, we observed that TCF-1 also has putative binding sites in *TNF-α*, *IL-17*, and *CSF2* promoters (Additional file 4: Figure S3 C). However, we did not observe the binding of TCF-1 to *CSF2* and *TNF*-α promoters in ChIP assay (data not shown). We did not observe IL-17 production in more than 50 % of healthy individuals. Together, these results suggest that TCF-1 binds to IFN-γ promoter and GZMB enhancer regions and regulates their expression in FTY720-activated T cells, and the effect of FTY720 on TNF-α and GMCSF expression does not depend on TCF-1.

### FTY720 downregulates Akt and GSK3 β phosphorylation and induces epigenetic modification

**Fig. 4:**
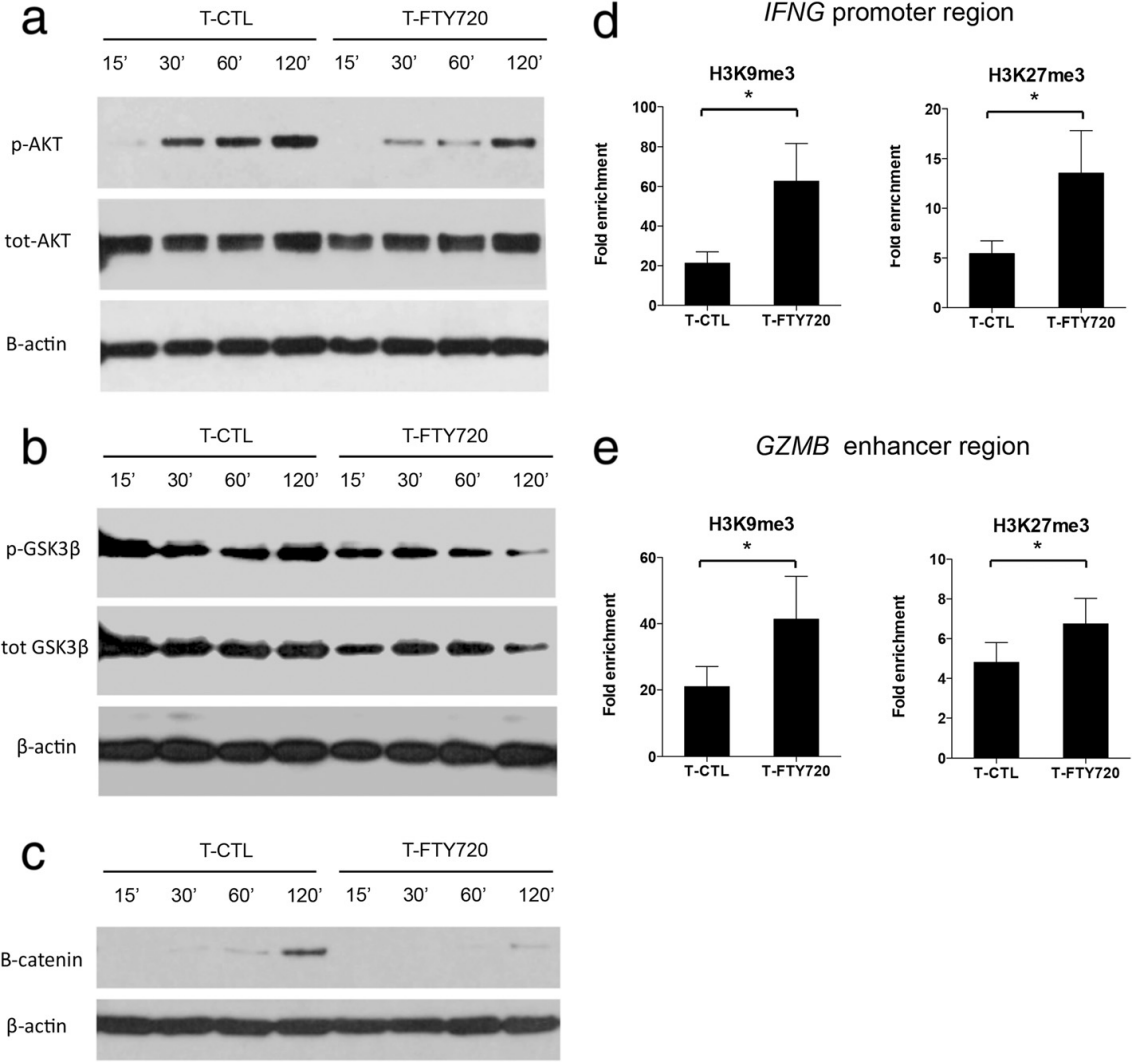
FTY720 downregulates Akt and GSK3β phosphorylation and induces epigenetic modification. **a** Western blot analysis of cell extracts from FTY720-treated (T-FTY720) and control T (T-CTL) cells at the indicated time points and analyzed for phosphorylated Akt (p-Akt) and total Akt (tot-Akt). β-actin served as a loading control. A representative image from one experiment is shown. **b** Immunoblot of phosphorylated GSK3β (pGSK3β) and total GSK3β (tot-GSK3β) in cell extracts from T-FTY720 and T-CTL cells at the indicated time points. β-actin served as a loading control. A representative image from one experiment is shown. **c** Immunoblot of β-catenin in cell extracts from FTY720 and T-CTL cells at the indicated time points. β-actin served as a loading control. A representative image from one experiment is shown. **d** qPCR analysis of chromatin-immunoprecipitated T cells showing methylation status of H3K9 and H3K27 on the IFNG promoter region (*n* = 7). Data are shown as mean ± SEM. *p < 0.05, paired non-parametric *t* tests. **e** qPCR analysis of chromatin-immunoprecipitated T cells showing the methylation status of H3K9 and H3K27 on the GZMB promoter region (*n* = 7). Data are shown as mean ± SEM. *p < 0.05, paired non-parametric *t* tests

Increased expression of TCF-1 upon FTY720 treatment suggests that perhaps FTY720 affects events downstream of S1P and Wnt signaling pathways. Phosphorylation of Akt, which is a fundamental step of the sphingosine pathway [39], induces phosphorylation of other proteins and enzymes such as GSK3β [34, 40]. Akt inhibits GSK3 kinase activity via phosphorylation of serine 9 (Ser9) in GSK3β, resulting in its inactivation [41, 42]. GSK3β also plays an important role in signaling downstream of the Wnt pathway. Inactivation of GSK3β allows unphosphorylated β-catenin to accumulate, translocate into the nucleus, and activate gene transcription by replacing co-repressor molecules bound to TCF-1 [43, 44]. On the other hand, decreased levels of phosphorylated/inactive GS3Kβ result in phosphorylation of β-catenin and its subsequent proteasomal degradation [43]. We performed western blot analysis to assess the phosphorylation status of Akt at Ser473 and of GSK3β at Ser9 in FTY720-treated and FTY720-untreated T cells at different time points. We found decreased phosphorylation of Akt at Ser473 (Fig. 4a) and GS3Kβ at Ser9 (Fig. 4b) in T-FTY720 cells compared with T-CTL cells at all time points. This reduced phosphorylated GS3Kβ associated with lower levels of β-catenin in T-FTY720 cells than in T-CTL cells (Fig. 4c). These results indicate that FTY720 inhibits β-catenin accumulation by reducing Akt-mediated inactivation/phosphorylation of GSK3β.

In the absence of β-catenin, target genes are silenced by TCF-1-mediated recruitment of TLE/Groucho proteins [45]. qPCR analysis showed no differences in the expression of *TLE1*, *TLE2*, and *TLE4* but a modest increase in *TLE3* expression in T-FTY720 cells compared with T-CTL cells (Additional file 5: Figure S4). The recruitment of TLE/Groucho proteins by TCF-1 results in transcriptional suppression of target genes by inducing epigenetic modifications [46, 47]. To gain further insight into the molecular mechanism by which FTY720-induced TCF-1 regulates IFN-γ and GZMB expression, we measured methylation status of histones H3K9 and H3K27 on the *IFNG* promoter and *GZMB* enhancer regions after FTY720 treatment using ChIP assay. We observed increased levels of tri-methylation at H3K9 and H3K27 in the *IFNG* promoter (Fig. 4d) and *GZMB* enhancer (Fig. 4e) regions in T-FTY720 cells compared with T-CTL cells. These results suggest that FTY720-induced TCF-1 regulates IFN-γ and GZMB expression by directly binding and inducing repressive chromatin modifications such as tri-methylation of Lys9 and Lys27 of histone H3.

### Expression of TCF7 is decreased in CD4+ T cells from multiple sclerosis patients

**Fig. 5:**
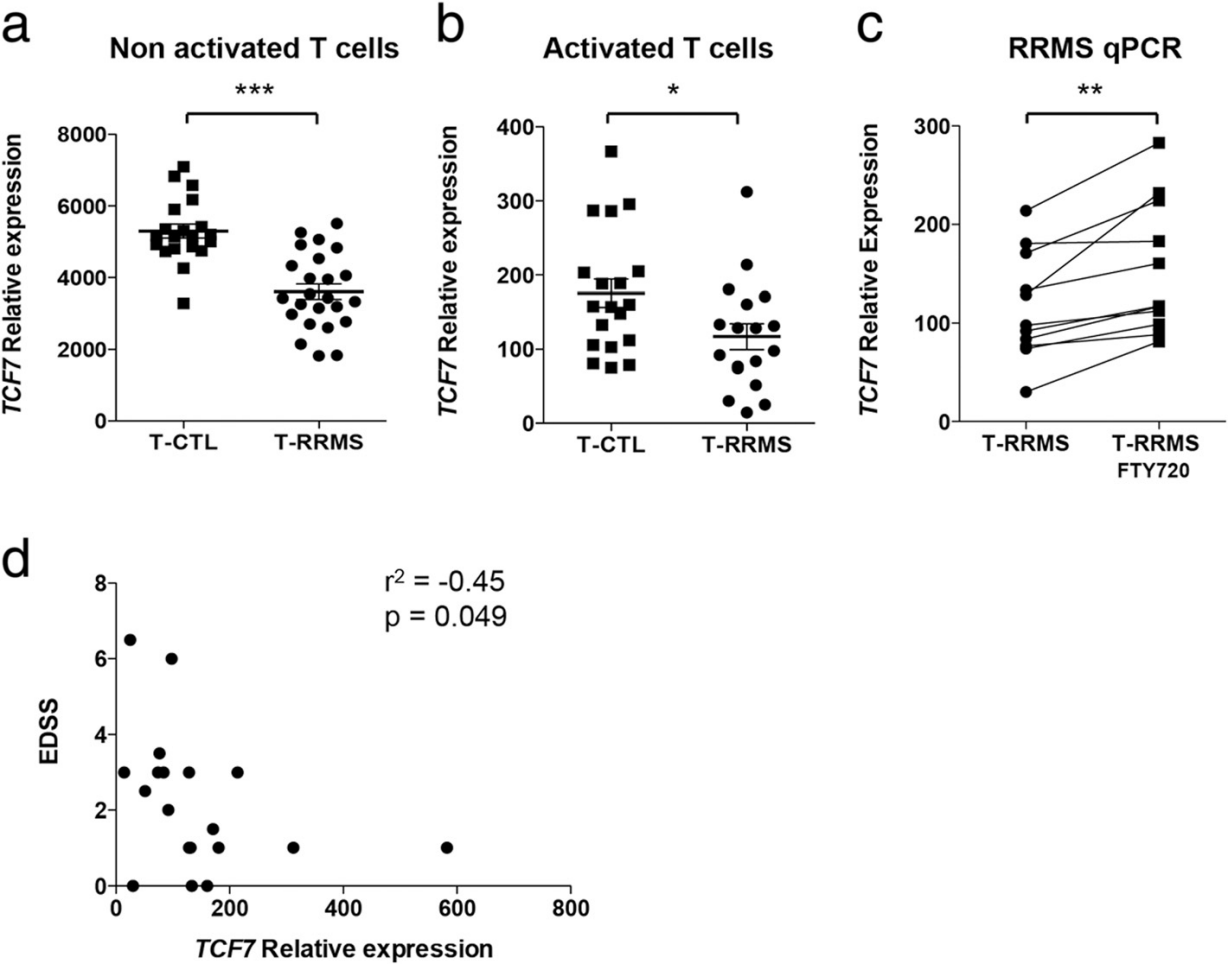
RRMS T cells show decreased *TCF7* expression. **a** Expression of *TCF7* in *ex vivo* T cells from RRMS patients (T-RRMS, *n* = 26) and healthy individuals (T-CTL, *n* = 21) measured using qPCR. **b** Expression of *TCF7* in activated T-RRMS cells (*n* = 18) and T-CTL cells (*n* = 19) measured using qPCR. **c** *TCF7* expression measured by qPCR in in vitro activated T cells from RRMS patients (*n* = 10) in the presence or absence of FTY720. **d** Correlation between TCF7 expression and EDSS score. *p < 0.05, **p < 0.01, ***p < 0.001, unpaired non-parametric *t* tests and Spearman correlation

To determine the relevance of TCF-1 in multiple sclerosis pathogenesis, we first assessed its expression in CD4+ T cells from multiple sclerosis patients. We found that *TCF7* expression was decreased in T cells from untreated RRMS patients (T-RRMS) compared with T cells from healthy controls (T-CTL) in both *ex vivo* isolated (Fig. 5a) and in vitro-activated (**b**) T cells. To assess whether FTY720 upregulates *TCF7* expression in T cells from RRMS patients, we activated T-RRMS cells in the presence or absence of FTY720 and found higher *TCF7* expression in FTY720-treated T-RRMS cells (Fig. 5c).

In order to determine whether the expression of *TCF7* is related to multiple sclerosis disease activity, we computed correlations between *TCF7* expression in T cells and clinical parameters such as Expanded Disability Status Scale (EDSS) score, disease duration, and number of relapses 2 years before the date of blood draw. We found a significant negative correlation (*r*^2^ = −0.45, *p* < 0.05) of *TCF7* expression with EDSS score (Fig. 5d). We did not find any correlations between *TCF7* expression and disease duration or number of relapses (data not shown). These results suggest that *TCF7* is involved in multiple sclerosis pathogenesis.

## Discussion

FTY720, the first oral drug approved for multiple sclerosis treatment, is an S1P analog that induces internalization of S1P receptors on T cells, resulting in lymphocyte sequestration inside lymph nodes [33]. In addition, FTY720 exhibits immunomodulatory effects in various immune cell populations [17, 31, 48–51], including T cells [52, 53], B cells [54], dendritic cells [55, 56], regulatory T cells [9, 57–60], and monocytes [56]. FTY720 also regulates the dichotomy between Th1 and iTregs through S1P_1_- and mTOR-dependent pathways [60]. Here, we examined the effect of FTY720 in human T cells and studied its role in the regulation of pathogenic effector T cell function in MS.

We found that FTY720 treatment increased *TCF7* expression and downregulated *IFNG* and *GZMB* expression in CD4+ T cells. These results are in line with a previous report which states that FTY720 treatment ameliorates EAE in mice and decreases IFN-γ and GZMB production in splenic CD8+ T cells [61].

In mice, TCF-1 expression is linked to regulation of inflammatory Th1 and Th17 differentiation and development of EAE [21, 36, 62]. In this study, we found that shRNA-mediated knockdown of *TCF-1* increases *IFNG* and *GZMB* expressions, indicating that TCF-1 may play a direct role in *IFNG* and *GZMB* expressions*.* Indeed, ChIP assay and reporter assay showed that TCF-1 not only directly binds to *IFNG* promoter and *GZMB* enhancer regions in FTY720-treated T cells but also regulates their expression. Although we found decreased expression of IL-17, GMCSF, and TNF-α upon FTY720 treatment, these cytokines were not followed further in the current study because IL-17 expression and production was not observed in more than 50 % of healthy individuals and ChIP assay did not detect binding of TCF-1 to *GMCSF* or *TNF*-α promoter regions (data not shown).

Tbet is a master regulator of Th1 differentiation that regulates TCF-1 expression through direct interaction with Bcl-6 [63]. However, we found no differences in the expression of *TBX21* or *BCL6* in FTY720-treated T cells (data not shown), suggesting that a different mechanism was involved in TCF-1 induction in this context. TCF-1 has also been shown to regulate Th2 differentiation through GATA3 in mice [19]. However, we found no changes in the expression of *GATA3* in human CD4+ T cells treated with FTY720 (data not shown).

Crosstalk between the sphingosine and Wnt signaling pathways has been previously shown in osteoblast-like cells through activation of Akt, GSK3β, and nuclear translocation of β-catenin followed by its association with TCF-1 [34]. In our study, we found decreased phosphorylation of Akt and GSK3β in FTY720-treated T cells and a reduction in β-catenin levels upon FTY720 treatment. Therefore, we speculate that in the presence of FTY720, there is a decrease in S1P_1_-mediated phosphorylation of Akt, which in turn decreases GSK3β phosphorylation. The active unphosphorylated GSK3β phosphorylates β-catenin, resulting in its proteasomal degradation. In the absence of β-catenin, TCF-1 binds to co-repressors such as TLE/Groucho family proteins and suppresses the transcription of target genes such as *IFNG* and *GZMB* (Additional file 6: Figure S5).

A recent mouse study shows that selective targeting of GSK3β in T cells leads to less severe EAE [63]. As the effect of GSK3β on TCF-1 expression was not investigated in this study, it is possible that therapeutic targeting of GSK3β might be linked to increased TCF-1 expression, which could reduce pathogenic T cell function and alleviate EAE symptoms.

In the absence of Wnt signaling, TCF-1 interacts with transcriptional repressor TLE/Groucho family members [62, 64], which repress transcription of target genes by inducing epigenetic modification [46, 47]. Acetylation and methylation on specific lysine residues are important epigenetic modifications for gene regulation. Transcription sites of actively transcribed genes are characterized by the presence of methylation at H3K4 or acetylation at H3K27, whereas gene repression is mediated by tri-methylation of H3K9 or H3K27 [65]. In mice, TCF-1 regulates Th17 differentiation through epigenetic changes [20, 21]. Here, we observed higher levels of histone H3K9 and H3K27 tri-methylation in the promoter region of *IFNG* and enhancer region of *GZMB* in T-FTY720 cells compared with T-CTL cells, supporting the hypothesis that FTY720 induces TCF-1-mediated regulation of IFN-γ and GZMB expression by chromatin modification. Although the mechanism of TCF-1 mediated epigenetic modifications was not addressed in this particular study, it is conceivable that TCF-1 brings about epigenetic changes by recruiting histone methyltransferases and other co-repressors such as TLE in the presence of FTY720.

Multiple sclerosis is the primary cause of non-traumatic neurologic disability in young adults. IFN-γ producing and GZMB secreting inflammatory T cells are implicated in its pathogenesis [3–6, 10, 66–68]. However, the regulatory mechanisms controlling pathogenic effector T cell function in multiple sclerosis are not completely understood. We found significantly decreased TCF7 expression in T cells from relapsing-remitting multiple sclerosis patients compared with those from healthy individuals and the treatment with FTY720 increased TCF-1 expression in both groups. Moreover, TCF7 expression in T cells is inversely correlated to disease disability as measured by EDSS.

## Conclusions

We observed that TCF-1 expression is reduced in T cells from relapsing-remitting patients. FTY720 treatment increases TCF-1 expression in CD4+ T cells from multiple sclerosis patients as well as from healthy individuals. The modulation of the sphingosine pathway is able to interfere with the downstream signaling of the Wnt pathway by reducing the availability of β-catenin and subsequently repressing the transcription of target genes. FTY720-induced TCF-1 expression in CD4+ T cells downregulates the pathogenic cytokines IFN-γ and GZMB by binding to their promoter/enhancer region and mediating epigenetic modifications. We also showed that *TCF7* expression negatively correlates with EDSS score in multiple sclerosis patients, suggesting a possible role of TCF-1 in the pathogenesis of multiple sclerosis. Together, our results reveal a previously unknown mechanism by which FTY720 functions in multiple sclerosis, and suggests that targeting TCF-1 signaling in CD4+ T cells could be a potential treatment strategy for multiple sclerosis.

## Additional files

Additional file 1: Table S1. Multiple sclerosis patient characteristics. All multiple sclerosis patients had relapsing-remitting disease courses. s.d. = standard deviation. Additional file 2: Figure S1. Flow cytometry analysis of CD4+ and naïve CD4+ T cells from multiple sclerosis patients before and after 3 months of treatment with fingolimod. BL = baseline, RV = revisit (*n* = 8). **p* < 0.05, ***p* < 0.01, paired non-parametric *t* tests. Additional file 3: Figure S2. FTY720 increases *TCF7* expression in T cells. Expression of *TCF7* in in vitro activated T cells from healthy individuals in the presence or absence of FTY720, natalizumab (T-Ty), or interferon beta (T-IFN beta). Data are shown as mean ± SEM. **p* < 0.05, ns = not significant, paired non-parametric *t* tests. Additional file 4: Figure S3. FTY720 reduces expression of pro-inflammatory cytokines. A) Expression of IL-17 (*n* = 9), GMCSF (*n* = 24), and TNFα (*n* = 24) in in vitro activated T cells from healthy individuals in the presence or absence of FTY720 using Luminex-based assay. (Note: IL-17 expression could not be measured in 15 healthy individuals.) B) Viabity staining of in vitro-activated T cells in the presence or absence of FTY720. Data are shown as mean ± SEM. **p* < 0.05, ****p* < 0.001, ns = not significant, paired non-parametric *t* tests. C) Binding region of TCF-1 in the promoter of *IL17*, *CSF2*, and *TNF-α*. Blue arrows represent transcription start site. Additional file 5: Figure S4. Effect of FTY720 on expression of TLE family proteins. Expression of *TLE1*, *TLE2*, *TLE3*, and *TLE4* using qPCR (*n* = 8). Data are shown as mean ± SEM. **p* < 0.05, ns = not significant, paired non-parametric *t* tests. Additional file 6: Figure S5. Proposed model of interaction between S1P_1_ and Wnt signaling pathways. A) S1P_1_ receptor activation induces Akt phosphorylation, which in turn induces phosphorylation of GSK3β. Phosphorylation of GSK3β at Ser9 leads to its inactivation, which allows unphosphorylated β-catenin to accumulate and translocate into the nucleus, where it binds to TCF-1 and induces transcription of target genes. B) In the presence of FTY720, the S1P_1_ receptor is downregulated, and less Akt is phosphorylated. Inactive Akt cannot phosphorylate GSK3β, which leads to the ability of GSK3β to phosphorylate β-catenin and thus, induce its degradation. In the absence of β-catenin, TCF-1 binds to co-repressors, such as TLE/Groucho family proteins, and represses the transcription of target genes.

## Abbreviations

CNS: central nervous system
EAE: experimental autoimmune encephalomyelitis
EDSS: expanded disability status scale
FTY720: fingolimod
GZMB: granzyme B
IFN-γ: interferon gamma
PBMCs: peripheral blood mononuclear cells
RLU: relative light units
RRMS: relapsing remitting multiple sclerosis
TCF-1: T cell factor 1
TNF-α: tumor necrosis factor alpha
