# Supplementary material for: Identification of a novel mechanism of action of fingolimod (FTY720) on human effector T cell function through TCF-1 upregulation
Source: J Neuroinflammation. 2015 Dec 30;12:245. doi: 10.1186/s12974-015-0460-z (PMC4696082; doi:10.1186/s12974-015-0460-z)
Supplement: Additional file 1: Table S1. — Multiple sclerosis patient characteristics. All multiple sclerosis patients had relapsing-remitting disease courses. s.d. = standard deviation. [file 12974_2015_460_MOESM1_ESM.pdf]

Supplementary table 1

**MS Patient's characteristics**	
Age in years [Mean (s.d.), range]	44 (±9.5), 26-54
Gender<br>Male [N (%)]<br>Female [N (%)]	 13 (27.6%)<br>34 (72.4%)
Disease duration [Mean (s.d.), range]	12.4 (±7.2), 1-34
EDSS [Mean (s.d.), range]	1.4 (±0.9), 0-6.5
