# Supplementary figures and images for: Identification of a novel mechanism of action of fingolimod (FTY720) on human effector T cell function through TCF-1 upregulation
Source: J Neuroinflammation. 2015 Dec 30;12:245. doi: 10.1186/s12974-015-0460-z (PMC4696082; doi:10.1186/s12974-015-0460-z)

# Supplementary Figure 1

A

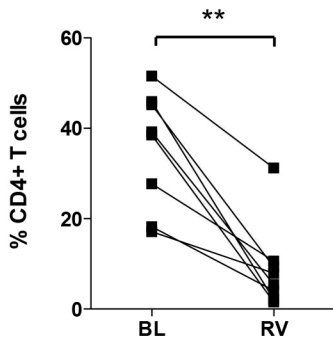

B

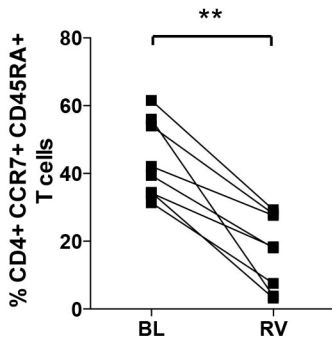

C

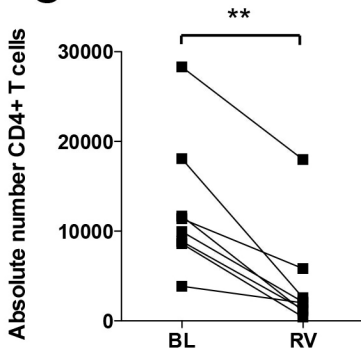

D

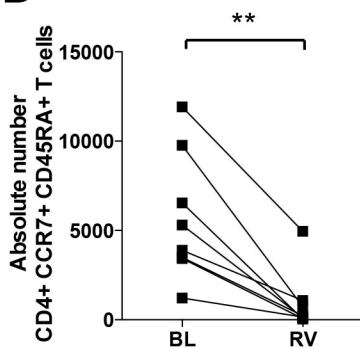

Supplement: Additional file 2: Figure S1. — Flow cytometry analysis of CD4+ and naïve CD4+ T cells from multiple sclerosis patients before and after 3 months of treatment with fingolimod. BL = baseline, RV = revisit (n = 8). *p < 0.05, **p < 0.01, paired non-parametric t tests. [file 12974_2015_460_MOESM2_ESM.pdf]

# Supplementary Figure 2

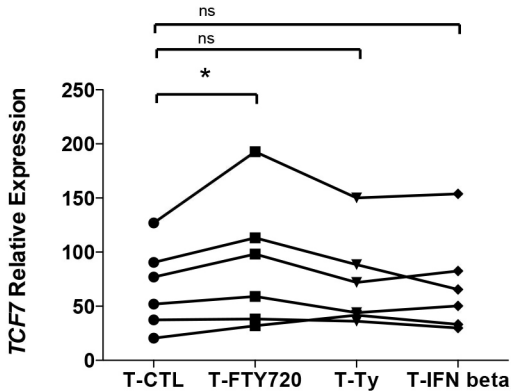

Supplement: Additional file 3: Figure S2. — FTY720 increases TCF7 expression in T cells. Expression of TCF7 in in vitro activated T cells from healthy individuals in the presence or absence of FTY720, natalizumab (T-Ty), or interferon beta (T-IFN beta). Data are shown as mean ± SEM. *p < 0.05, ns = not significant, paired non-parametric t tests. [file 12974_2015_460_MOESM3_ESM.pdf]

# Supplementary Figure 3

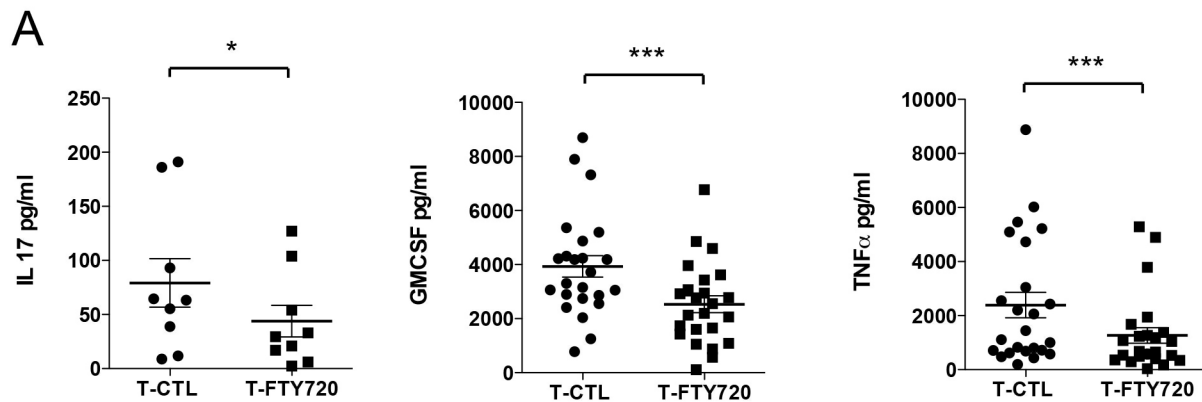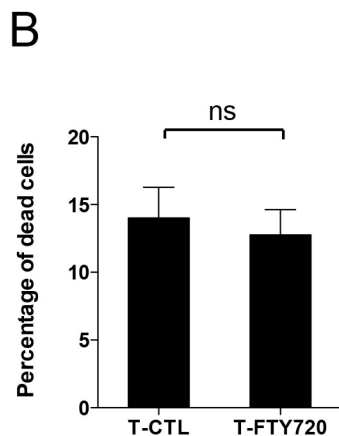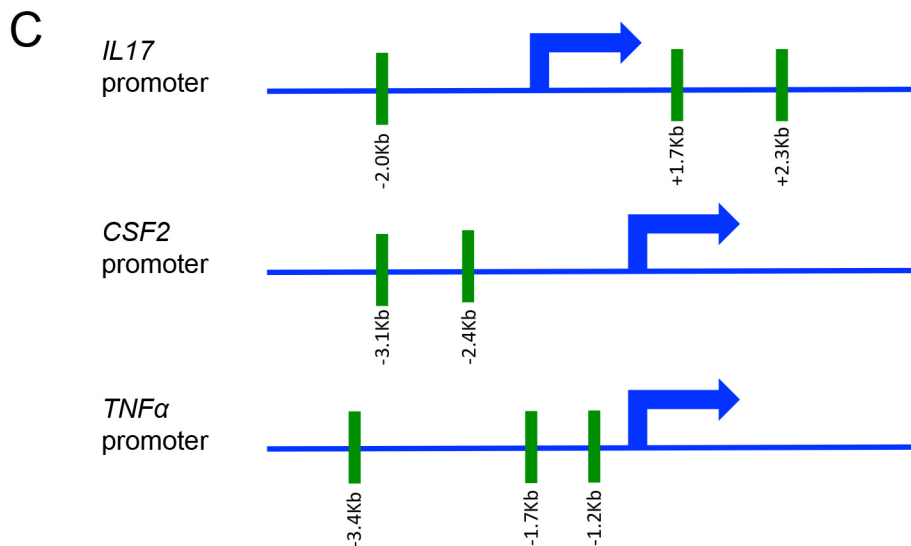

Supplement: Additional file 4: Figure S3. — FTY720 reduces expression of pro-inflammatory cytokines. A) Expression of IL-17 (n = 9), GMCSF (n = 24), and TNFα (n = 24) in in vitro activated T cells from healthy individuals in the presence or absence of FTY720 using Luminex-based assay. (Note: IL-17 expression could not be measured in 15 healthy individuals.) B) Viabity staining of in vitro-activated T cells in the presence or absence of FTY720. Data are shown as mean ± SEM. *p < 0.05, ***p < 0.001, ns = not significant, paired non-parametric t tests. C) Binding region of TCF-1 in the promoter of IL17, CSF2, and TNF-α. Blue arrows represent transcription start site. [file 12974_2015_460_MOESM4_ESM.pdf]

## Supplementary Figure 4

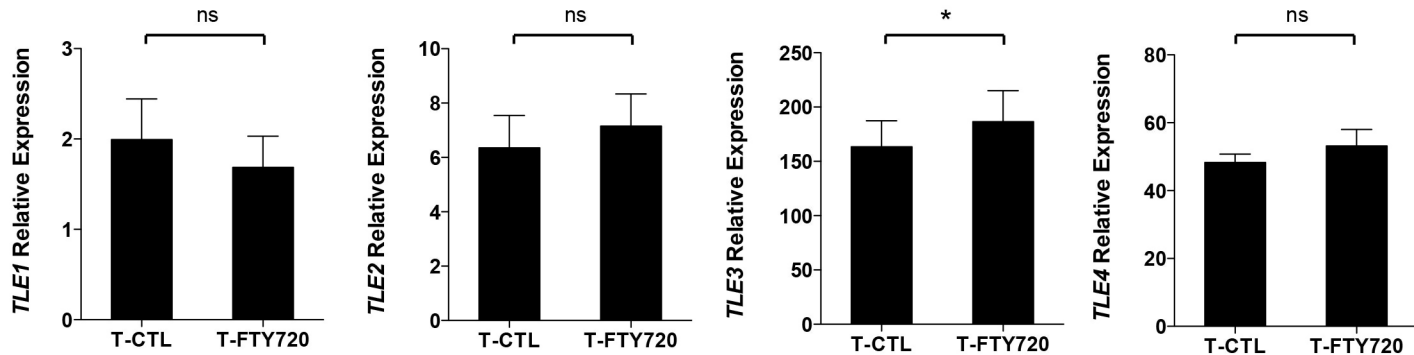

Supplement: Additional file 5: Figure S4. — Effect of FTY720 on expression of TLE family proteins. Expression of TLE1, TLE2, TLE3, and TLE4 using qPCR (n = 8). Data are shown as mean ± SEM. *p < 0.05, ns = not significant, paired non-parametric t tests. [file 12974_2015_460_MOESM5_ESM.pdf]

Supplementary Figure 5

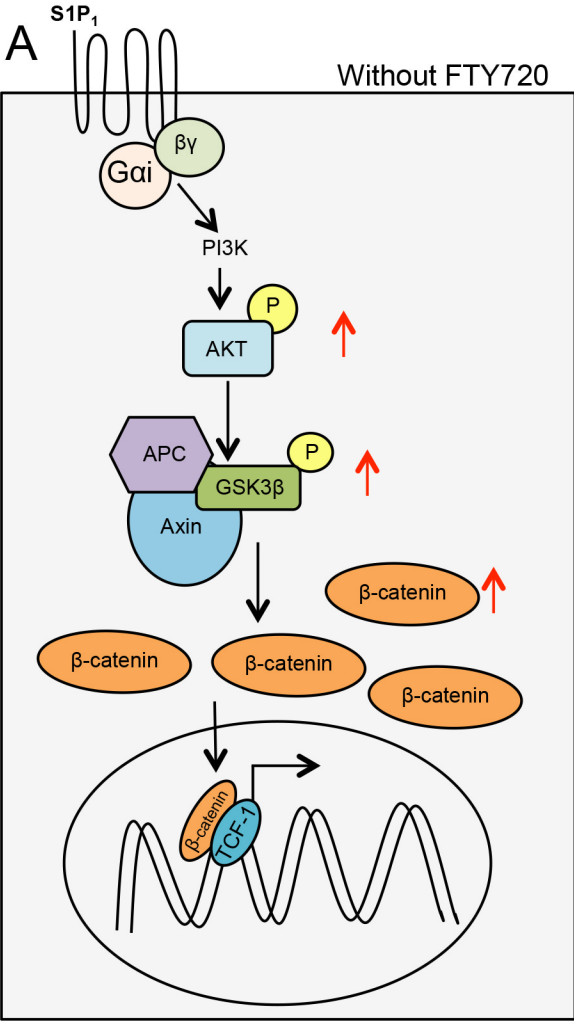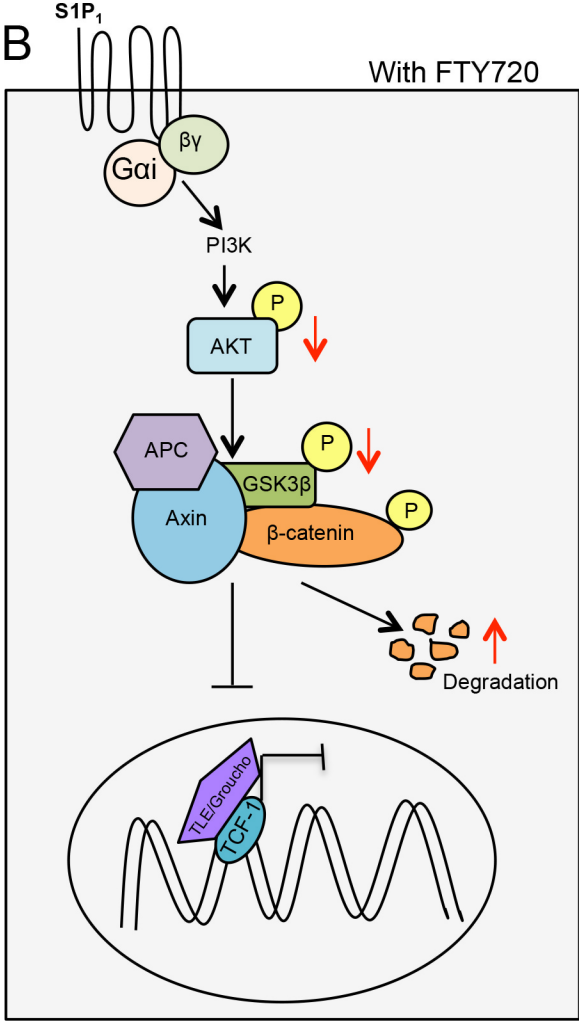

Supplement: Additional file 6: Figure S5. — Proposed model of interaction between S1P1 and Wnt signaling pathways. A) S1P1 receptor activation induces Akt phosphorylation, which in turn induces phosphorylation of GSK3β. Phosphorylation of GSK3β at Ser9 leads to its inactivation, which allows unphosphorylated β-catenin to accumulate and translocate into the nucleus, where it binds to TCF-1 and induces transcription of target genes. B) In the presence of FTY720, the S1P1 receptor is downregulated, and less Akt is phosphorylated. Inactive Akt cannot phosphorylate GSK3β, which leads to the ability of GSK3β to phosphorylate β-catenin and thus, induce its degradation. In the absence of β-catenin, TCF-1 binds to co-repressors, such as TLE/Groucho family proteins, and represses the transcription of target genes. [file 12974_2015_460_MOESM6_ESM.pdf]
